# Are there gender based differences in participation and time spent in physical activity in Albania? Evidence from 2017-18 demographic and health survey

**DOI:** 10.1186/s13690-022-00930-2

**Published:** 2022-08-11

**Authors:** Monika Çule, Harminder Guliani

**Affiliations:** Department of Economics, University of Regina, Regina, Saskatchewan Canada

**Keywords:** Physical activity, Socioeconomic factors, Double hurdle model, Gender differences, Albania

## Abstract

**Background:**

Since 1990, Albania has embraced the market economy and globalization. Prosperity and modernization have also brought significant lifestyle changes toward unhealthy behaviours, doubling the mortality from non-communicable diseases (NCDs). While Physical Activity (PA) can mitigate the NCDs burden, participation is low in Albania. To date, research on PA determinants that could meaningfully inform policy is lacking. To fill this gap, this study examines the PA behaviour among Albanian adults. Specifically, we assess the decisions to participate, and the time spent in PA.

**Methods:**

Using the 2017–2018 Albania Demographic and Health Survey data and a double-hurdle regression model, we simultaneously assess the influence of demographic, socioeconomic and lifestyle factors on the likelihood of participating (extensive margin) and the time spent (intensive margin) in PA. To understand gender differences regarding PA decisions, we run separate models for men and women.

**Results:**

Results show significant variations in the likelihood of participation and the time spent in PA, by household economic status, administrative regions, occupation, and education. We find that likelihood of participation in PA increases with household wealth, but conditional on participation, affluent Albanians spent less time in PA. Education and employment status also have opposite effects on participation and time spent margins. Results show notable gender-based differences in PA behaviour (either for participation or time spent) related to education, age, family structure (marital status and the number of young children), regions, occupation, and lifestyle factors.

**Conclusions:**

Insights in understanding the PA behaviour of Albanian adults allow policymakers to identify socio-demographic groups most in need of intervention effort. To effectively support PA among Albanians, policymakers should target males and females differently and address gender-specific needs accordingly.

**Supplementary Information:**

The online version contains supplementary material available at 10.1186/s13690-022-00930-2.

## Background

Health benefits of Physical Activity (PA) in reducing the risk of all-cause mortality, the cardiovascular mortality, and morbidity are well established [1, 2]. Regular PA among adults is beneficial in preventing and reducing the risk of several non-communicable diseases (NCDs) such as cardiovascular disease (heart, stroke, and hypertension), type-2 diabetes, and various cancers, which account for 71% of all deaths worldwide [3, 4]. In addition, PA benefits the brain and mental health by improving cognition, reducing anxiety, reducing the risk of depression and dementia, and improving sleep. PA is also important for achieving and maintaining a healthy weight, improving physical functioning, and bone health. Furthermore, PA is particularly beneficial to older adults as it lowers the risk of falls and fall-related injuries [3].

These health benefits have become critically apparent during the Covid-19 pandemic. NCDs comorbidities and pre-existing conditions of cardiovascular and metabolic diseases considerably increased the risk of morbidity and mortality among Covid-19 patients ([5–7], among many others). Furthermore, lifestyle changes associated with lockdowns and remote working and learning created conditions for increased sedentary behaviours. Deliberate PA has therefore become even more imperative in protecting health. For instance, [8] find that PA has played an important protective role against poor mental health during the pandemic.

In 2020, the World Health Organization (WHO) updated its guidelines for participation in PA. To achieve health benefits, the WHO recommends at least 150 to 300 minutes of moderate aerobic activity per week (or the equivalent of vigorous intensity) for adults and 60 minutes of moderate aerobic PA per day for children and youth [1]. The 2020 guidelines remove the minimum 10 minutes bouts of exercise required previously and emphasize that any PA under the recommended levels is more beneficial than none. Hence, any PA participation in any form and duration is highly encouraged [9]. This shift in approach aligns with behavioral science in supporting people’s psychological needs, motivation and ability to participate in PA [10]. The new approach aims at enticing people to integrate and incrementally increase their physical activities in their daily lives, which in turn could be more effective in achieving PA levels that realize the above health benefits.

Despite the known health benefits of PA, the share of population that achieve the recommended level is relatively low. Global estimates found in [11], where 27.5% of adults did not meet the guidelines in 2016, indicate that no improvements were observed during the last two decades on the global participation rates in PA [9]. However, the inactive population percentages vary across the group of low-income, middle-income, and high-income countries, with 15.5%, 27.5% and 31.1% of adult populations reported to be inactive, respectively [12]. Furthermore, in a few high-income countries, low levels of adult participation in PA are much more concerning. For instance, in 2016, among U.S. adults, only 26% of men, and 19% of women, reported sufficient PA to meet the guidelines [3].

Given the concerning state of physical inactivity worldwide, WHO launched the Global Action Plan on PA in 2018 aiming to reduce the physical inactivity by 10% by 2025 and by 15% by 2030 [13]. While the Global Action Plan provides a set of multipronged strategies for countries to address the problem, it is incumbent upon national governments to develop and implement comprehensive national policies for PA. A 2019 WHO survey revealed that only 40% of countries have national guidelines regarding PA, and only 32% have operational policies. Within the European region, where Albania, the country of interest in this study belongs, 60% of countries have national guidelines, with only 53% having operational policies [4].

Unfortunately, Albania is not one of them. Since 1990, Albania has undergone unprecedented transformations by democratizing the political system, developing a market-based economy, and embracing globalization. While benefits resulting from political freedoms, economic prosperity, and modernization are undisputed, they have also been accompanied by significant lifestyle changes toward unhealthy behaviours. The NCDs and mortality burden have been steadily rising in recent decades [14, 15]. In 2010, lifestyle risk factors accounted for 70% of the total disease burden in Albania.

Alarmingly, overall mortality rates due to various risk factors have more than doubled over the 1990–2010 period [15]. Specifically, the mortality rate due to overweight and obesity more than doubled, with 2.5 times increase from heart disease and 3 times increase from diabetes. The death rate from heart disease from hypertension more than doubled while the one from stroke due to hypertension increased by 70%; Unhealthy diet, obesity, physical inactivity have contributed to a doubling of mortality rate from heart disease and a tripling of mortality rate from diabetes [15].

Although PA is an important mitigating factor in reducing NCDs burdens, physical inactivity in Albania is relatively high. According to the 2017–2018 Albania Demographic and Health Survey (ADHS), 69% of the adult population (18–59 years) does not engage in any PA, with 64% of men and 71% of women, respectively.^1^ The deteriorating health status of the Albanian population and the high levels of physical inactivity beg urgent policy actions to address the concerning trends regarding NCDs.

This study examines the contributing socioeconomic, demographic, and lifestyle factors on participation and time spent on PA in Albania. Our work is informed by numerous studies that examine the socioeconomic and demographic correlates of PA in terms of participation decision, the time spent, as well as the frequency and/or the intensity of PA in various countries. While the measure of participation in PA and sports may vary depending on the available data, many cross-sectional studies find that participation in PA is affected by age, sex, educational attainment, household income, family structure in terms of marital status and number of dependent children of various ages, employment status, ethnicity, race and regions.

Consistently, studies find a positive association between educational attainment and participation in PA [16–26]. In addition, many studies find that men are more likely to exercise and engage in PA and sports than women [17, 20, 21, 24, 26], but others find the opposite [18, 19, 27, 28]. Several studies that perform a stratified by gender analysis [16, 17, 21, 22, 25, 29] reveal gender differences on the effects of socioeconomic factors on PA. In addition, most studies find that participation in PA increases with income [16, 22, 25, 27, 30], but declines with age [17, 19, 21, 23, 25, 28, 30, 31]. Generally, being married is negatively associated with PA participation [16, 19, 21, 23, 25, 27, 29], although some find the opposite effect [20, 28, 31]. Having school-age children is also negatively associated with PA participation, particularly for women [16, 20, 22, 26, 29, 30].

In the health economics literature, there is a particular focus on the role of income/wages as it directly relates to the opportunity cost of engaging in leisure-time PA, thus capturing the trade-offs of intensity and duration of PA undertaken [16, 17, 27, 29, 31]. Additionally, a few studies examine PA decisions on participation and time spent margins [17, 19, 21, 22, 24, 27, 28], which reveal a more nuanced story with a number of determinants sometimes affecting PA behaviours in opposite ways. For instance, while income positively affects participation, it negatively affects the time spent on PA [18, 19]. Being married, gender and age also have a more complex association with PA on these different margins [18, 19, 22]. Finally, a number of studies include other lifestyle factors such as smoking and alcohol consumption in the analysis to capture the clustering effect of health risk behaviours [16, 20, 21, 23, 28]. While smoking is mostly found to be negatively associated with PA participation, drinking is positively related and thus does not act as a barrier to participating in PA.

Using the 2017–2018 Albania Demographic and Health Survey data (ADHS), we consider a number of socioeconomic, demographic, and lifestyle factors that affect the participation and time spent in PA of the adult population (aged 18 to 59 years). Using the double hurdle regression model, we explore three questions. First, who is at risk of physical inactivity in Albania? Second, are there any differences in factors that influence the decision to participate and the time spent in PA? Furthermore, are there gender-based differences regarding these factors?

This study contributes to the above-mentioned literature in two ways. First, to our knowledge, this is the first and only study that focuses on the PA determinants in Albania using national-level data to understand who is at risk of being physically inactive and not realizing health benefits from PA.^2^ Our results provide important insights for policymakers to design a targeted policy that could most effectively increase the level of PA among various socio-demographic groups of the Albanian population.

Secondly, while there is a considerable body of literature that examines the determinants/correlates of PA and sports participation in the high-income country context (for instance, US [17, 19], Canada [18], UK [20, 23], Scotland [21], Australia [16, 27, 29], Spain [22], Austria [25] among others), studies for mid-income and developing countries are much more limited. Malaysia has received attention by Chea and others [24, 28, 31], Brazil in [30] and Taiwan in [26]. With Albania being a mid-income country, this study adds to the latter body of work.

## Methods

### Data source

We used 2017–18 ADHS data conducted by the Institute of Statistics and the Institute of Public Health with technical assistance from Inner City Fund (ICF) international and funded by international agencies. The DHS is a large-scale, cross-sectional household survey that uses a multistage cluster sample design and provides information on population and health issues on nationally representative samples of males and females. Various researchers have assessed the quality of DHS surveys for different health indicators, such as maternal mortality and nutritional status and found them to be highly reliable [33, 34]. The DHS manual includes details on 2017–18 ADHS samples [35]. The overall response rate for ADHS data is relatively high - 95% for households, 93% for women, and 87% for males [35]. The survey collected respondents’ information on various socioeconomic and demographic factors at the national and regional levels, including gender, age, education, wealth index, occupation, and marital status. The survey also included data on health indicators such as respondent’s general health status, lifestyle indicators such as participation in PA, tobacco smoking and alcohol drinking. Our analysis sample contains 19,052 adults between the ages of 18–59 years, of whom 13,652 are females and 5400 are males.

### Econometric analysis

Engaging in PA is a two-part decision-making process: first, the choice to participate at all and second, the time spent in PA [18, 19, 36]. For instance, an individual must first decide to take a walk or ride a bike. Second, having made this decision, the individual must determine how long to walk or bike and/or how frequently to do so. While these are separate choices, they are related. As such, we model the factors affecting participation in PA both at the extensive (participation decision) and intensive (time spent decision) margins. Humphryes and Ruseski (2011) thoroughly describe the conceptual economic framework on participation and time spent in PA [19].

We utilize Cragg’s double hurdle model [36] to empirically evaluate whether certain factors influence individuals’ decisions about PA differently on the extensive and the intensive margins. Specifically, we presume that a group of factors influencing the decision to participate in PA (the first hurdle) may differ from those that determine the time (the second hurdle) that a potential participant will eventually spend on PA. These two ‘hurdles’ must be overcome before observing a positive outcome [36].

Following Jones (1989) [37], we estimate the likelihood of participation and time spent in PA using the following specifications:

Observed equation: *y* = *d* × *y* ∗  ∗ 

Participation equation: p = α^′^z + μ μ~N(0, 1)

$$\mathrm{d}=\left\{\begin{array}{cc}1& \mathrm{if}\ \mathrm{p}>0\\ {}0& \mathrm{otherwise}\end{array}\right.$$

Time spent equation: $${\displaystyle \begin{array}{cc}{\mathrm{y}}^{\ast }={\upbeta}^{\prime}\mathrm{x}+\upvarepsilon & \upvarepsilon \sim \mathrm{N}\ \left(0,{\upsigma}^2\right)\end{array}}$$

$${\mathrm{y}}^{\ast \ast }=\left\{\begin{array}{cc}{\mathrm{y}}^{\ast }& \mathrm{if}\ \mathrm{d}=1\kern0.5em \mathrm{and}\ {\mathrm{y}}^{\ast }>0\\ {}0& \mathrm{otherwise}\end{array}\right.$$

Where *z* and *x* are a set of factors influencing participation and time spent decisions, respectively. Our analyses specify a probit model form to assess the influence of factors affecting a decision to participate *(p)* and an exponential form for determining factors influencing the time spent active (y*), conditional on participation. The parameters of both equations are estimated simultaneously using maximum likelihood. The error terms of both equations *(μ* and *ε)* are assumed to be normal and independently distributed.

The double hurdle approach has been extensively applied in various behaviours involving a two-part decision-making process such as sports participation, tobacco consumption, alcohol consumption, and gambling [18, 19, 37–39]. Further, hurdle models provide the ability to account for a large number of zeros and positive skewness that may be observed in the data [37]. Zeros in data may be due to deliberate abstentions (the corner solution to a constrained utility maximization problem) or non-observable responses due to missing or non-responses [18, 19, 40]. These non-responses could be due to the survey design (the time frame for the survey was too short of including the behavior, for instance) [18, 19]. About 64% of individuals in our sample reported not participating in any PA. We conjecture those zeros in our data result from an optimal choice and not due to non-observable responses since the DHS elicited individual responses on participation in PA in a usual week.

The alternate empirical models for handling non-participation are Tobit and Heckman sample selection model. The Tobit model is too restrictive as it assumes all the zeros to be the respondents’ deliberate choices. Moreover, the probability of participation and the duration of time spent in PA decisions are simultaneously influenced by the same set of explanatory variables. We test the Tobit/double hurdle choice in our data using a standard likelihood ratio test since the Tobit model is nested in double hurdle under the hypothesis that the parameters on both equations are identical. A large, computed value of test statistic from our data (LR = 12,965.8 with associated *p*-value< 0.001) strongly rejects Tobit specification favouring the double hurdle model.

The Heckman selection model (also referred to as Tobit II) treats zeros in the selection model as cases of unobserved or missing data and assumes dependence between the participation and time spent decisions. Jones (1989) points out that the Heckman model assumes first hurdle dominance, i.e., the participation decision dominates the time spent decision - ruling out corner solutions of the utility maximization problem [37].

Nonetheless, we compare the double hurdle model and the sample selection model in our data using Vuong’s (1989) modified likelihood ratio test for non-nested maximum likelihood estimators [18, 37, 41]. Test results indicate that both models can be considered equivalents. However, Buraimo et al. (2010) suggested that the double hurdle approach offers more reliable estimates than Tobit or the Heckman sample selection model [38]. The double hurdle model is also less restricted than Heckman and best suited for random samples like ours [35, 42].

To account for possible correlation between unobservable factors affecting participation and time spent decisions, we initially estimated the “full double hurdle” model [18, 37]. Following Angel and Moffat (2014), we tested for the possible correlation of error terms between the participation and time spent equations using the inverse-mills ratio in the spirit of Heckman’s two-step estimator [43]. The statistically not significant coefficient obtained suggests that error terms are indeed not correlated. We, therefore, report estimates from the double hurdle models with independence in error terms of both equations.

To improve the identification of parameters in estimating double hurdle models, following Humphreys and Ruseski (2015), we excluded two variables from the time-spent equation – the household’s access to motor vehicles and the individual’s health status compared to a year ago [18]. The choice of exclusion variables is often made arbitrarily, and therefore, we seek guidance from the existing theoretical and empirical literature [18, 23]. Having no access to a car, truck or motorbike may likely negatively influence the first hurdle decision of participation, particularly in leisure PA occurring in sports facilities in distant locations. On the other hand, having no access to a vehicle could positively influence participation as individuals choose more physically active ways of transportation (walking or biking) in their daily activities. However, once the first hurdle is passed, having no vehicle will likely not influence the time spent. Similarly, following [18], the improved health status compared to a year ago is more likely to influence an individual’s decision to participate in PA, but improvements in health status may not affect the time spent decision.

Lastly, we split the sample by gender and fit separate models for men and women to look for gender differences regarding PA decisions. We use sampling weights from the men’s and women’s files to adjust for the unequal probability of selection [44]. Standard errors are adjusted for potential misspecification errors, such as non-normality and heteroskedasticity. Average marginal effects are calculated to understand the magnitude of the relationship. STATA version 15 is used for all data analyses.

### Study variables

#### Dependent variables

We define participation in PA using two survey questions: 1) “In a usual week, do you do activities such as walking, bicycling, jogging, or other things that increase your breathing and heart rate?”; and 2) “How do you usually go to work every day, walking, riding a bicycle or by other means of transportation?” We create a binary variable, “participation in any PA,” which equals one if respondents answered yes to question 1 above and/or also walked or rode a bike to their work. This binary variable takes the value 0 if the respondent did not do active recreation or used other means of transportation to work. WHO Global Action Plan encourages integrating all forms of PA into settings in which people live, work, and play [13]. Motivated by this, we combine leisure-time and active time commuting to work. A 15-minute bike ride, for instance, would provide the same health benefits, whether it is for leisure or commuting to the workplace.

The Albanian DHS asked further questions about how much time (in minutes) participants spent per day doing physical activities. The related questions on the time spent and the frequency of participation are: 1) “On the days when you engage in these activities, how much time in total do you usually spend doing these activities?”; 2) “How many days per week do you do these activities?”; 3) “Normally, how long does it take you to go to work (walking /bicycling) every day?” We use the answers to these three survey questions to assess the total time (in minutes) spent participating in PA per week. For instance, if a respondent reported spending 30 minutes per day doing leisure time activities every day and 10 minutes of biking or walking to work (assuming 5 days of work), their total time participating in PA worked out to be 260 minutes per week (30*7 + 10*5).

#### Independent variables

We include several socioeconomic and demographic covariates in modelling factors affecting participation and time spent in PA. The existing literature on factors affecting the decision to participate in PA guided the choice of these variables [17–23, 45]. These factors include age categories in years; education levels; household economic status, marital status; urban/rural place of residence; 12 administrative regions; occupation status; the number of preschool and school-age children in the household; and lifestyle factors such as smoking, drinking habits, and healthy eating habits.

Household economic status is measured by the DHS’s wealth index, which is a composite measure of a household’s cumulative living standard. The DHS calculates the wealth index using household ownership of assets and consumer items such as televisions, bicycles, cars, and dwelling characteristics, including materials used for housing construction, source of drinking water and sanitation facilities. Each household is then classified into quintiles where the first quintile is the poorest 20% of households, and the fifth quintile is the wealthiest 20%.

Smoking status takes the value of one if respondent smoked tobacco in any form such as cigarettes, chewing tobacco, cigar or any country-specific. Moderate drinking refers to males having two drinks or women having one drink, on one occasion, on the days they drink. Similarly, fruits and vegetable consumption is a dummy variable equal to one if the respondent consumed recommended servings of veggies (4 or more) and fruits (3 or more) per day.

As noted above in the econometric analysis section, the participating equation includes two additional variables as per exclusion restrictions: respondent’s self-reported health status compared to year-ago; and household access to a motor vehicle (car/truck/scooter). Table 1 provides summary statistics of the dependent and independent variables used in the study.

**Table 1:** Summary Statistics for the dependent and independent variables, 2017–18 Demographic and Health Survey, Albania (*N* = 19,052)

Variables	Mean/%	Standard Deviation
**Participation in any PA**	0.313	0.464
**Minutes spent per week, conditional on participation**	168.652	155.496
**Gender**		
Male	0.283	0.451
**Education Level**		
No education/Primary less than 4-year	0.022	0.145
Primary 8-year	0.455	0.498
Secondary/Professional/Technical	0.355	0.478
University and Post-graduate	0.169	0.375
**Age**		
18–24	0.158	0.364
25–29	0.108	0.311
30–34	0.102	0.303
35–39	0.099	0.299
40–44	0.110	0.313
45–49	0.128	0.334
50–54	0.143	0.350
55–59	0.152	0.359
**Marital Status**		
Never married	0.192	0.394
Currently Married or living together	0.767	0.423
Divorced/separated/ widowed	0.040	0.197
**Occupation Status**		
Unemployed	0.564	0.496
Professional/technical/managerial	0.077	0.267
Clerical	0.014	0.117
Sales and Services	0.068	0.252
Skilled manual	0.094	0.291
Unskilled manual	0.088	0.283
Agriculture	0.096	0.294
**Lifestyle variables**		
Smoking	0.126	0.332
Moderate Drinking	0.262	0.440
Healthy eating habits	0.048	0.215
**Health Status compared to a year ago**		
Better	0.280	0.449
Same	0.631	0.483
Poor or worse	0.089	0.286
**Regions**		
Berat	0.072	0.259
Dibër	0.100	0.294
Durrës	0.080	0.275
Elbasan	0.082	0.275
Fier	0.090	0.286
Gjirokastër	0.068	0.252
Korçë	0.098	0.298
Kukës	0.091	0.287
Lezhë	0.067	0.250
Shkodër	0.080	0.272
Tiranë	0.106	0.908
Vlorë	0.067	0.251
**Household economic status**		
Very Poor / Poor	0.514	0.500
Middle	0.195	0.397
Very Rich/Rich	0.291	0.454
**Household access to motor vehicle**	0.469	0.499
**Number of young children in the household (0–4 years)**	0.257	0.534
**Number of school-age going children in the household (5–15 years)**	0.537	0.817
**Place of residence**		
Urban	0.466	0.499

For continuous variables, the values refer to mean and standard deviation. For categorical variables, percentages and standard deviations are reported; Estimates are unweighted

## Results

### Descriptive

**Fig. 1:**
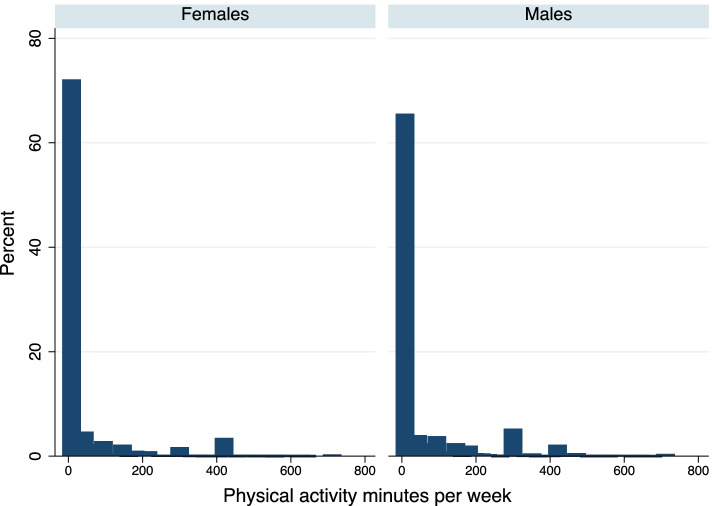
Distribution of minutes spent per week on physical activity by gender, 2017–18 Demographic and Health Survey, Albania

Figure 1 reports the unweighted frequency distribution of minutes spent per week in any PA separately for males and females. These distributions show excessive zeros and positive skewness. Table 2 reports the weighted estimates. As seen, 60% of males and 67% of females reported zero involvement in any PA. Conditional on participation, males and females spent on average 185 and 175 minutes per week, respectively, which is within the WHO recommendation.

**Table 2:** Physical activity by participants’ socioeconomic and demographic characteristics stratified by gender, 2017–18 Demographic and Health Survey, Albania^a^

	Participation (%)		Minutes spent per week	
	***Males***	***Females***	***Males***	***Females***
**Average**	40.41	33.62	184.98	175.1
**Household economic status**				
Very poor/Poor	37.27	27.24	182.92	174.13
Middle	36.08	33.26	154.54	163.75
Rich/very rich	45.24	39.82	197.79	180.3
**Education**				
No education/ Primary less than 4-year	25.77	20.21	249.49	153.82
Primary 8-year	38.37	28.13	181.55	164.64
Secondary/Professional/Technical	40.52	36.02	182.33	164.65
University/Post-graduate	45.15	41.25	192.2	201.74
**Regions**				
Berat	57.52	44.85	167.35	104.21
Dibër	24.78	24.56	228.44	256.51
Durrës	32.06	18.26	118.30	94.18
Elbasan	31.11	35.86	222.33	155.82
Fier	51.05	34.35	108.28	168.14
Gjirokastër	23.51	35.01	164.59	169.52
Korçë	24.96	40.47	111.70	142.70
Kukës	28.54	16.82	296.40	155.87
Lezhë	38.47	19.08	123.74	122.22
Shkodër	38.35	17.71	177.56	106.37
Tiranë	47.75	41.47	229.58	213.69
Vlorë	47.78	38.33	203.09	190.59
**Occupation**				
Unemployed	20.01	15.13	182.86	241.23
Professional/technical/managerial	47.68	55.09	229.61	185.42
Clerical	65.18	35.76	225.2	173.13
Sales and Services	52.3	60.82	182.96	138.52
Skilled manual	42.8	47.99	186.82	121.61
Unskilled manual	50.92	57.11	161.15	154.38
Agriculture	61.43	72.86	172.58	145.57

^a^Weighted estimates

Table 2 reveals considerable gender differences in participation and time spent in PA across selected socioeconomic and demographic groups. Rich Albanian men and women are more likely to be physically active than their poor counterparts, with differences being statistically significant. There exists an education-based gradient in PA participation with higher education leading to an increased level of participation. Males residing in Berat, Fier, Tiranë and Vlorë show a higher level of PA (48–58%) than males in other regions. Females in Berat, Korçë and Tiranë regions have higher levels of participation (40–45%) than females in other regions. Unemployed men and women are less active than their employed counterparts. Women employed in a professional field, sales and services, and agriculture appear to have a higher level of PA participation than their male counterparts, with these gender differences being statistically significant.

Table 2 also reports differences in average time spent per week, conditional on participation. A similar pattern is noticeable in wealth, with rich males and females being more likely to spend on average more time in PA per week than their poor counterparts. Conditional on participation, males with no or less than 4 years of primary education are likely to spend on average more time per week than those with university and postgraduate level of education. While fewer unemployed women participate in PA, those who do, spend on average more time in PA per week than employed ones.

### Regression results

Table 3 reports estimation results from the double hurdle model for males and females separately. We report average marginal effects (AMEs) with a 95% confidence interval (CI) in parentheses. Depending on the definition of the expected value of the dependent variable, we can calculate three different marginal effects from the double hurdle estimates: a) AME for the probability of any participation; b) AME for the unconditional expected mean of the average minutes spent per week – i.e., an overall effect including those who do not participate; c) AME for the conditional mean of the average minutes spent per week, conditional on participation. We report the marginal effects for the probability of any participation and the average minutes spent per week conditional on participation. The overall effect on average minutes per week is provided in Additional file 1. Overall, the model fits the data well, as shown by the result of the Wald test for overall significance. In what follows, we discuss and interpret results on variables that are significant.

**Table 3:** Estimates from Double-hurdle regression model for physical activity participation and the amount of time spent by gender, 2017–18 Demographic and Health Survey, Albania

	Males		Females	
	Participation	Time spent (Mins. per week)	Participation	Time spent (Mins. per week)
	AME (95% CI)	AME (95% CI)	AME (95% CI)	AME (95% CI)
**Education Level (Ref. University and Post graduate)**				
No education/Primary less than 4-year	−0.077 (− 0.216, 0.062)	78.485* (− 9.843, 166.813)	− 0.142*** (− 0.218, − 0.065)	−29.274 (− 97.075, 38.527)
Primary 8-year	−0.032 (− 0.106, 0.042)	21.866 (− 20.223, 63.956)	− 0.031 (− 0.069, 0.006)	−31.745* (−66.412, 2.923)
Secondary/Professional/Technical	− 0.020 (− 0.079, 0.038)	20.007 (−16.083, 56.098)	0.008 (− 0.026, 0.041)	−38.075** (−70.319, −5.831)
**Age (Ref:** 18-24 years)				
25–29	− 0.071** (− 0.135, − 0.006)	−2.511 (− 44.429, 39.407)	0.007 (− 0.032, 0.046)	−10.924 (− 54.884, 33.036)
30–34	− 0.122*** (− 0.192, − 0.052)	−37.906 (−90.476, 14.663)	0.005 (− 0.044, 0.054)	1.407 (− 42.104, 44.917)
35–39	− 0.081* (− 0.174, 0.011)	−11.625 (− 65.978, 42.727)	0.037 (− 0.015, 0.089)	−15.080 (− 61.481, 31.320)
40–44	− 0.089** (− 0.176, − 0.003)	16.752 (− 46.173, 79.677)	0.064*** (0.018, 0.110)	−17.280 (−61.059, 26.500)
45–49	− 0.046 (− 0.125, 0.033)	−4.552 (−61.670, 52.567)	0.057** (0.009, 0.105)	−13.123 (− 57.398, 31.152)
50–54	− 0.090** (− 0.171, − 0.009)	− 23.197 (−82.029, 35.636)	0.055** (0.006, 0.105)	−10.382 (−53.206, 32.442)
55–59	−0.050 (− 0.140, 0.040)	−6.400 (−62.521, 49.721)	0.046* (− 0.003, 0.094)	−8.840 (− 52.498, 34.819)
**Marital Status (Ref: Never Married)**				
Currently Married or living together	− 0.060* (− 0.127, 0.008)	−0.650 (− 43.817, 42.516)	−0.064*** (− 0.102, − 0.026)	−14.618 (− 50.595, 21.358)
Divorced/separated/ widowed	−0.032 (− 0.163, 0.099)	42.603 (−33.454, 118.660)	−0.036 (− 0.092, 0.020)	−39.670* (− 84.730, 5.391)
**Occupation Status (Ref: Unemployed)**				
Professional/technical/managerial	0.268*** (0.178, 0.358)	84.172*** (31.831, 136.514)	0.294*** (0.247, 0.342)	−75.520*** (− 112.207, − 38.833)
Clerical	0.395*** (0.250, 0.541)	84.919*** (24.251, 145.588)	0.154*** (0.087, 0.221)	−100.470*** (− 168.849, −32.091)
Sales and Services	0.315*** (0.254, 0.377)	22.585 (−24.435, 69.604)	0.357*** (0.318, 0.395)	−139.441*** (− 174.487, − 104.395)
Skilled Manual	0.268*** (0.209, 0.326)	32.978 (− 10.464, 76.421)	0.289*** (0.244, 0.334)	− 104.618*** (− 139.192, − 70.044)
Unskilled Manual	0.348*** (0.285, 0.412)	4.384 (−41.963, 50.732)	0.326*** (0.289, 0.363)	−112.180*** (− 147.753, −76.606)
Agriculture	0.478*** (0.413, 0.543)	50.576** (7.575, 93.577)	0.491*** (0.452, 0.529)	−70.776*** (−103.060, − 38.491)
**Lifestyle variables**				
Smoking	−0.027 (− 0.067, 0.013)	−2.817 (− 25.841, 20.207)	− 0.009 (− 0.065, 0.046)	54.799*** (15.467, 94.130)
Moderate Drinking	0.005 (− 0.043, 0.053)	−15.049 (−39.800, 9.703)	0.028* (− 0.001, 0.058)	9.749 (− 10.612, 30.110)
Healthy eating habits	0.011 (− 0.100, 0.121)	63.648** (9.674, 117.623)	0.045* (− 0.006, 0.096)	14.168 (− 23.572, 51.908)
**Health Status compared to a year ago (Ref: Better)**				
Same	− 0.050 (− 0.114, 0.014)		− 0.101*** (− 0.130, − 0.072)	
Poor or worse	−0.054 (− 0.145, 0.038)		−0.017 (− 0.058, 0.024)	
**Regions (Ref: Tiranë)**				
Berat	0.046 (−0.037, 0.129)	−101.667*** (− 147.724, −55.609)	0.039 (− 0.015, 0.093)	−157.231*** (− 189.095, − 125.367)
Dibër	−0.174*** (− 0.260, − 0.089)	−21.344 (−68.567, 25.880)	0.035 (− 0.032, 0.103)	20.397 (− 12.674, 53.467)
Durrës.	−0.105** (− 0.200, − 0.011)	−79.256*** (− 118.376, − 40.137)	−0.161*** (− 0.219, − 0.103)	−179.532*** (− 225.787, − 133.277)
Elbasan	−0.133** (− 0.254, − 0.012)	−58.397* (− 125.879, 9.085)	0.021 (− 0.034, 0.077)	− 89.327*** (− 121.893, −56.761)
Fier	−0.008 (− 0.093, 0.077)	− 144.920*** (− 183.681, − 106.159)	−0.038 (− 0.094, 0.018)	−60.539*** (−91.144, − 29.933)
Gjirokastër	−0.225*** (− 0.332, − 0.118)	−131.150*** (− 203.876, − 58.425)	0.011 (− 0.059, 0.080)	−53.951*** (−86.110, − 21.791)
Korçë	−0.282*** (− 0.384, − 0.181)	−191.043*** (− 246.664, − 135.422)	−0.040 (− 0.095, 0.015)	−89.227*** (− 121.135, − 57.319)
Kukës	−0.101* (− 0.222, 0.019)	75.603*** (36.167, 115.039)	−0.142*** (− 0.219, − 0.064)	− 82.356*** (− 120.794, − 43.919)
Lezhë	−0.030 (− 0.129, 0.068)	−115.384*** (− 158.318, − 72.449)	−0.090*** (− 0.153, − 0.028)	−100.030*** (− 144.886, − 55.174)
Shkodër.	−0.048 (− 0.146, 0.049)	− 90.524*** (− 135.948, − 45.100)	−0.120*** (− 0.176, − 0.065)	−137.663*** (− 175.876, − 99.449)
Vlorë	0.003 (− 0.084, 0.091)	−25.175 (− 66.974, 16.625)	0.037 (−0.037, 0.111)	− 55.514*** (− 90.668, − 20.360)
**Household Economic Status (Ref: Very Poor/ Poor)**				
Middle	0.012 (− 0.041, 0.065)	− 51.881*** (− 85.446, − 18.317)	0.031** (0.004, 0.059)	− 32.819** (− 60.269, −5.369)
Rich/Very Rich	0.059** (0.004, 0.115)	− 35.090* (− 71.736, 1.556)	0.031* (− 0.003, 0.065)	−42.737*** (− 70.917, − 14.558)
**Household access to motor vehicle**	−0.052** (− 0.095, − 0.009)		−0.008 (− 0.033, 0.017)	
**Number of young children in the household (0–4 yrs)**	− 0.020 (− 0.061, 0.021)	−44.055*** (− 74.787, − 13.323)	−0.026** (− 0.048, − 0.004)	−11.518 (− 31.043, 8.008)
**Number of school going children in the household (5–14 yrs)**	− 0.011 (− 0.040, 0.018)	4.783 (−12.919, 22.485)	0.008 (−0.008, 0.023)	1.756 (−12.194, 15.706)
**Place of residence (Ref: Rural)**	−0.047 (− 0.105, 0.011)	9.333 (− 21.084, 39.749)	0.023 (− 0.015, 0.061)	19.860 (−4.601, 44.321)
**Wald Chi2 (*P*** **> Chi2)**	378.20(0.00)		493.12 (0.00)	
**Log pseudolikelihood**	−14,921.809		−30,949.493	
**Number of observations**	5400		13,652	

*AME* Average Marginal effects, *CI* Confidence interval

*** *p*-value < 0.01, **p-value < 0.05, *p-value < 0.10

We find that females with less than primary education are 14% less likely to participate in PA than their university-educated counterparts (Table 3). Education does not statistically influence the males’ PA participation. However, we found the opposite effects of gender on the intensive margin. Conditional on participation, Albanian women with primary and secondary education spend 32 and 38 fewer minutes per week, respectively, than those with university education. In contrast, conditional on participation, males with no/less than primary education spend on average 78.5 more minutes per week in PA than their university-educated counterparts. Although similar results can be seen for primary and secondary levels of education, they are statistically not significant.

Age is found to be a statistically significant predictor for the likelihood of participation but does not significantly associate with the time spent in PA. While older males are less likely to participate than young male adults (18-24 years), females behave differently. Females older than 40 years are more likely to participate compared to females 18-24 years of age. Marital status is significantly associated with the likelihood of participation for both males and females, though results are highly significant for females. Married men and women are 6 % less likely to participate in any PA. Given the participation, formerly married and widowed women spend on average 39.7 fewer minutes per week than unmarried women. The number of young children (0–4 years) has a statistically negative influence on the decision to participate for females and on the time spent for males.

Compared to unemployed Albanians, employed adults are more likely to participate in any PA regardless of gender. The extent varies by occupation, with males and females employed in agriculture being 48 and 49%, respectively, more likely to participate in PA. However, we find contrasting results on the time-spent margin by gender. Physically active females, employed in all sectors, spend on average less time (70–139 minutes per week) in PA, while males employed across some sectors spend more time in PA compared to their unemployed counterparts.

Among lifestyle factors, smoking does not significantly affect PA participation for both genders, but physically active females who smoke spend more time (55 minutes per week) in PA. Women who moderately consume alcohol and have healthy eating habits are three and 4 %, respectively, more likely to participate in PA. Males with healthy eating habits spend more time (63.6 minutes per week) in PA. The change in health status statistically influences participation for females only. Females who perceive the same health status compared to a year ago are 10% less likely to participate in PA than those who perceive having better health.

Compared to Tirana residents, adults residing in other regions, where we obtained statistically significant results, are less likely to participate in PA. Additionally, except for Kukës males, residents of other regions spend less time in PA than Tirana residents. The magnitude of time spent results varies considerably by regions and by gender. For instance, Elbasan and Korçë males spend 58 and 191 fewer minutes per week, respectively, than Tirana males. Gjirokastër and Durrës females spend 54 and 180 fewer minutes per week, respectively, than Tirana females.

Rich and very rich Albanian males and females are more likely to participate in PA than their poor and very poor counterparts, by six and 3 %, respectively. However, we find a negative effect on the time-spent decision. Conditional on participation, Albanian males and females in top wealth quintiles spend 35 and 43 fewer minutes per week in PA, respectively, than those in the bottom two quintiles. Similarly, middle-income males and females spend 52 and 33 fewer minutes, respectively, than their poor and very poor counterparts.

Household access to car/truck/scooter has a negative association with the participation rate for males only. Males who have access to a vehicle are 5 % less likely to participate in PA than those who do not.

Our results are robust to alternate model specifications. For instance, we estimate the model by including additional variables such as the BMI status of individuals in both participation and time spent equations and the current self-reported health status in the time spent equation. While the coefficients of BMI status and current health status are statistically not significant, we obtain qualitatively similar results on all other socioeconomic and demographic variables.

## Discussion

Our findings about the PA behaviour of Albanian adults are generally consistent with theoretical expectations and the existing literature on the determinants of PA in other high or medium-income countries. We start our discussion with the effect of household economic status on PA behaviours.

### Household economic status

Our results on economic status are in line with those reported in other countries that show that likelihood of participation in PA increases with income [16–22, 25, 27, 30]. Since cash-rich individuals place increased value on healthy days, the wealthy individuals are better able to allocate more money to PA participation, such as purchasing a gym membership or buying home exercise equipment, including bikes, treadmills, etc.

However, similar to [18, 19, 24], we find that conditional on participation, men and women in high-income households spent on average less time in PA. This result is more pronounced for females, with rich females spending 43 fewer minutes per week in PA than poor women. Since PA participation requires inputs of both time and market goods, the opportunity cost of time spent on producing health increases for high-income individuals, affecting the optimal frequency and duration of PA. Time-poor, high-income individuals may be substituting market inputs (e.g., health care, healthy diet) for their own time to achieve a given level of health stock [19, 31].^3^ Due to the higher opportunity cost of time, high-income individuals may engage in vigorous (higher) intensity but shorter duration exercise in order to achieve the same health benefits [17, 27, 31]. We have no information on the intensity of the PA undertaken and therefore are not able to judge whether the shorter time spent in PA may still achieve the same health outcome for the rich individuals.

### Gender-based differences in PA behaviours

Our results show few notable gender-based differences in PA behaviour (either for participation or time spent) as it relates to education, age, family structure, regions, employment/occupation, and lifestyle factors. We now turn our attention to discussing these factors.

### Educational attainment

As argued in [46], well-educated people appear to be better informed about the health benefits of being active and face fewer financial constraints than less-educated people. Moreover, highly educated people tend to have one steady job affording them more time for leisure PA, which, in return, reduces the real cost of PA services (i.e., gym memberships/classes). In contrast, low-income individuals likely hold two or more low paying jobs, a situation that not only limits their leisure time PA but also makes PA opportunities less affordable in real terms. As reported earlier, empirical studies have consistently validated the positive relationship between educational attainment and high participation in PA [16–26].

Similarly, we find that women with less than primary years of education are less likely to participate and spend less time in PA activities than university-educated counterparts. Education does not statistically influence the males PA participation, but it influences the time spent margin in an opposite way compared to females. This may suggest a rising opportunity cost of time for highly educated males, which plays out on the intensive margin of PA.

### Age

Our results show that age correlates differently with PA participation across genders, while there is no statistically significant effect of age on the time spent. Consistently with other studies that show participation in PA declines with age [17, 19, 21, 23–25, 28, 30, 31], we find that older males are less likely to participate compared to young male adults (18-24 years). In contrast, females older than 40 years are more likely to participate in PA than young females, with no significant effects found for those in the 25–29, 30–34, 35–39 years age groups. Our result for females may suggest that women older than 40 years may face fewer barriers to participating in PA that could well relate to childrearing responsibilities within the household, which are more common for women younger than 40. Few studies have shown a nonlinear (or U-shaped) relationship of PA participation with age [18, 22, 27]. Our finding about females appears to partially fit the notion that the propensity to participate in PA increases as people approach the mid-age and retirement age.

### Family structure

Consistent with other studies [16, 19, 21, 23, 25, 27, 29], our results indicate that married couples are less likely to participate in PA than single Albanians. This result aligns with the notion that the additional non-market work associated with marriage increases the opportunity cost of participating in PA. Another possible explanation relates to the single person’s youthful motivation to be physically fit and attractive while searching for a partner in the marriage market, a motivation married people no longer have.

Although being married has no statistically significant effect on time spent for both genders, our results reveal another gender-based difference for the formerly married adults. Unlike men, physically active women who are separated/divorced/widowed spend on average 39.7 fewer minutes per week in PA than singles. These women may face additional barriers that could be related to their child (ren) and managing households on their own, therefore allocating less time in PA.

In the existing literature, the presence of school-age children is negatively associated with participation in PA [16, 20, 22, 26, 29, 30]. We find that the presence of young children lowers the likelihood of participation only for women, thus pointing to another gender-based difference. On the intense margin, while there is no statistically significant effect on time spent in PA for women, physically active men spend less time in PA. A possible explanation for this different pattern across genders could be linked to their role in childcare. Traditionally, mothers are the primary caregivers in Albania, while fathers play a supporting role. Hence, mothers must often forgo their PA altogether to attend to their children, while fathers only reduce their time spent in PA.

### Employment/occupation status

Compared to unemployed Albanians, employed adults are more likely to participate in PA regardless of gender, a finding that is in line with others [16, 20, 25, 28, 29, 31]. One plausible explanation could be that employed individuals use active transportation modes to get to work, a measure accounted for in our PA variable. Also, employed individuals may choose to partake in PA to cope with the stress and pressures of work. Additionally, in the professional world, physical appearance and fitness has historically been believed to be positively correlated to the propensity for job promotions, raises and general career success. Employed, therefore, would have a higher propensity to participate in PA. Lastly, employed individuals may likely be more aware of PA benefits due to workplace health promotion programs or may have access to workplace gym memberships, although these are less common in Albania.

However, we note another gender difference on the intensive margin of the effect of occupation. Employed physically active females spend on average less time in PA, while males employed across some sectors spend more time in PA compared to their unemployed counterparts. Two points are worth noting here. First, the result may be driven by the high share (81%) of females among the unemployed in our sample. One could expect that unemployed, physically active females have greater flexibility in allocating their leisure time and hence, spending more time in PA. Second, the opportunity cost of time may dominate financial constraints on the intensive margin for employed females, as shown in [29, 31]. Working mothers must juggle both market and non-market responsibilities and thus, may not be able to spend a lot of time in PA. Like the higher burden young children place on women, working females face a higher opportunity cost of time compared to males.

There is a recurring theme in our results regarding the different effects of income, education, age, family structure and employment/occupation status on PA behaviours across genders. These gender-based differences may be explained by the higher burden of childcare and other non-market household responsibilities that women face, which in turn adversely affect their inclination to participate and the time spent in PA. They point out the need for policymakers to target males and females differently as they take action to encourage and support a higher level of PA.

For instance, specific efforts targeting less-educated females with educational campaigns to increase awareness about PA health benefits could promote both their participation and an adequate level of PA. Additionally, PA programs in the workplace could effectively increase the time spent in PA for time-poor working women. Such programs require the participation of employers. Given the competitive labour market conditions in Albania,^4^ it is difficult to expect that private sector employers would offer additional non-monetary benefits, including PA workplace programs, at a level observed in advanced economies. Nevertheless, since the national, regional, and local governments in Albania are major employers, this policy action could be impactful if implemented in the public sector workplaces.

### Regional and lifestyle factors

There are a few points worth noting regarding the regional results. First, the general result that Tirana residents are more likely to participate and spend more time in PA is likely driven by Tirana’s advantage in economic and educational status, both of which have a positive effect on PA behaviours.

Secondly, within each region, there are noticeable gender differences in PA behaviours compared to Tirana counterparts. For instance, Korça males are 28% less likely to participate and, conditional on participation, spend 191 fewer minutes in PA than Tirana males. There is no significant effect on participation for Korça females, but physically active Korça females have a lower time deficit (89 minutes per week). In Shkodër, the reverse pattern is observed for males. This wide regional variation across genders implies a need to target the female and male populations within the local context. It would make sense that PA promotion efforts to increase PA participation should target more aggressively males in Korçë but females in Shkodër.

Some regional variations may be related to built environments in many Albanian cities and towns pertaining to their natural situation. Cities located on the seaside, lakeshore, foothills or mountainous areas would typically provide their residents with better opportunities for physical activities such as swimming, uphill walking, hiking, etc. Given that built environments within each city would provide its residents with the same opportunities to engage in PA, regardless of gender, one would expect that all else equal, there would be little to no gender difference in the PA behaviours within a region.

The gender differences we observe could reflect the point that males and females perceive the built environment differently as it relates to their PA behaviour. A systematic literature review [48] identifying built environmental determinants of PA accounting for sex/gender suggests that easy access to public transport and safety in cycling lanes are critical considerations for females' PA behaviour. On the other hand, street network characteristics such as intersection connectivity are more important considerations among men [48].

Our results on lifestyle factors are generally in line with findings of other studies that consider the clustering effect of risky health behaviours. We note another gender-based difference as it relates to lifestyle factors and health status. While moderate drinking, healthy eating and improved health status show no statistically significant effect on males’ participation in PA, they positively associate with female PA participation. A similar result regarding moderate drinking is found for young adult women in Australia [16]. Authors explain this positive association based on reports from other studies that find moderate drinking to be associated with a lower risk for diabetes and cardiovascular conditions [16].

In addition, we find that although smoking does not significantly affect PA participation for both genders, the physically active females who smoke spend more time in PA. This result appears counterintuitive to what is expected, whereas having a high time discount, smokers invest less in healthy behaviours and what is found in [20, 21, 23, 26, 28].

However, as shown in [49], in post-communist Albania, smoking is often viewed as a sign of female emancipation, leading to an increase in female smoking prevalence in the last decades. The richer and more educated females are more likely to smoke tobacco compared to their poor and uneducated counterparts. Physically active female smokers (more likely to be rich and well educated) perhaps spend more time in PA to reduce the harmful health effects of smoking. In our sample, 45.8% of female smokers belong to the top two wealth quintiles and 36.1% had university and/ post-graduate level of education.

### Access to a vehicle and other discussion points

Lastly, we discuss the effect of access to a vehicle on participation. We find that having access to a vehicle is negatively associated with PA participation for males only, reflecting the common observation that Albanian households would typically have access to a single vehicle, which is primarily driven by males. Studies that considered this factor in the UK and Scotland context found the opposite [20, 21]. The lack of vehicles would make commuting to sports/recreation facilities more difficult and thus, impede PA participation.

Our finding can be explained by the fact that such facilities are not commonplace in Albania. Also, recall that in addition to leisure-time, our PA measure includes the walking/biking time to work. Those with a vehicle use this sedentary means of transportation and forgo some PA. In our sample, only 28.3% of those with a vehicle choose to walk/bike to work. Perhaps most pursue the active commute not necessarily for health benefits but because they have no other means of transportation. This result provides a warning for the NCDs concerning trends in the last two decades.

Beyond our results, we pose another related discussion point regarding the changing lifestyles and its implication for PA and sedentary behavior in Albania. Until 1990 private car ownership was nonexistent in Albania. Most relied on walking/biking to carry out their daily activities, including commuting to workplaces. With little noise and emission pollution and significantly more pedestrian-friendly cities, active commuting was a much more enjoyable experience than it is today. Furthermore, the fast-paced and ill-planned urban expansion has resulted in inadequate green, recreational and pedestrian-friendly spaces in major cities like Tirana [50]. Active commute was still the dominant form of transportation a decade ago. The 2011 Census data showed that 50.2% of the employed population used walking/biking as a means to commute to the workplace [51]. However, this is changing. With higher living standards and the rising car ownership, the Albanian population is increasingly relying on more sedentary means of transportation for daily activities. Meanwhile, WHO recommends that any form of PA in any setting is beneficial and maintaining an active commute is important. To integrate physically active commute by choice, people need to be supported with adequate and safe infrastructure.

Additionally, structural changes in the economy point towards sectors that increasingly require less physically demanding occupations. For instance, while the share of employment in the services sector increased from 34.4% in 2000 to 43.4% in 2019, the share of employment in agriculture declined from 52.8 to 36.4% [47].

With further economic development, as the population shifts away from organically incorporating PA in daily work activities, people would need to pursue PA more proactively through leisure and sports participation. This in turn, requires sports and recreational infrastructure and an urban expansion with adequate built environments that facilitate walking/biking and other forms of physical activities. A national action plan for PA should deliberately incorporate support for such infrastructure.

The study has a few limitations, and therefore our findings should be interpreted with some caution. First, given the subjective nature of the PA measurement, our estimates may be subject to reporting and recall bias. Second, PA is assumed of moderate intensity due to data limitations, and some respondents have likely engaged in vigorous PA. Moreover, data constraints further preclude us from capturing specific work-related PA, which is likely to affect health differently. Third, as mentioned before, our results establish the association among various factors and PA and in no way reflect a causal effect.

## Conclusions

Using 2017–2018 ADHS data, this study examines the contributing socioeconomic, demographic, and lifestyle factors on participation and time spent in PA for Albanian adults. The gender-stratified analysis shows that the likelihood of participation in PA increases with income, but conditional on participation, rich and very rich men and women spend less time in PA. Education and employment status also have opposite effects on participation and time spent margins.

Furthermore, there are notable gender-based differences in PA behaviour (either for participation or time spent) as it relates to education, age, family structure in terms of marital status and number of young children, regions, employment/occupation, and lifestyle factors. These gender differences may be explained by the higher burden of childcare and other non-market household responsibilities that women face, which in turn adversely affect their inclination to participate, and the time spent in PA.

Given the concerning trend of rising mortality and morbidity from NCDs, there is an urgency for the Albanian government to mitigate the problem by developing and implementing a comprehensive national policy on PA. While the 2018 Global Action Plan on PA provides a blueprint for various strategies to scale up action at the national level [13], local conditions will dictate, to some extent, the feasibility of policy responses. Our results provide additional and valuable insights for understanding the PA behaviour of Albanian adults and identifying target groups most in need of intervention effort. Most importantly, to effectively promote and support both the uptake and higher levels of PA in Albania, policymakers need to target males and females differently and address gender-specific needs accordingly.

## Supplementary Information

**Additional file 1: Supplementary Table 1.** Estimates from Double-hurdle model: minutes spent per week (the unconditional mean), 2017–18 Demographic and Health Survey, Albania.

## Data Availability

The data is available upon request through USAID’s The DHS Program at: https://dhsprogram.com/data/dataset/Albania_Standard-DHS_2017.cfm?flag=1

## Abbreviations

ADHS: Albania Demographic and Health Surveys
NCD: Non-communicable disease
PA: Physical Activity
WHO: World Health Organization

## Footnotes

1 Authors’ calculations from the DHS data [unweighted estimates] used in this study, available at https://dhsprogram.com/data/dataset/Albania_Standard-DHS_2017.cfm?flag=1.

2 This is understandable since Albania DHS 2017–2018 is the first national level survey that inquires information about the participants’ involvement in PA. Melonashi and Shkembi [32] is the only study in the Albanian context that explicitly considers a PA variable among other lifestyle behaviors. However, their specific focus on risky behaviours of the university student population, provides limited insights for the population at large.

3 Our data does not permit constructing a wage variable, a better proxy for measuring the opportunity cost of time [19]. However, as [19] suggest, the household income and education are positively correlated with wages and their coefficients can be used as a proxy for the omitted wage variable.

4 The World Bank Development Indicators for Albania report an unemployment rate of 11.5% in 2019. The labor force participation rate was 60.3%, with a much lower rate in the youngest population group (15–24 years of age) at 36.6%. Women share in the labor force was 43% [47].
