# Supplementary material for: Are there gender based differences in participation and time spent in physical activity in Albania? Evidence from 2017-18 demographic and health survey
Source: Arch Public Health. 2022 Aug 11;80:187. doi: 10.1186/s13690-022-00930-2 (PMC9366130; doi:10.1186/s13690-022-00930-2)
Supplement: Supplementary file 1 — Additional file 1: Supplementary Table 1. Estimates from Double-hurdle model: minutes spent per week (the unconditional mean), 2017–18 Demographic and Health Survey, Albania. [file 13690_2022_930_MOESM1_ESM.docx]

| **Supplementary Table 1: Estimates from Double-hurdle model: minutes spent per week (the unconditional mean), 2017-18 Demographic and Health Survey, Albania** | | | |
| --- | --- | --- | --- |
|  | Male | | Female |
|  | AME  (95% CI) |  | AME  (95% CI) |
| **Education Level (Ref. University and Post graduate)** | | | |
| No education/Primary less than 4-year | 18.065  (-29.514, 65.646) |  | -38.141***  (-63.167, -13.114) |
| Primary 8-year | 2.916  (-19.318, 25.150) |  | -16.163**  (-29.542 -2.785) |
| Secondary/Professional/Technical | 4.433  (-15.240, 24.108) |  | -10.083  (-22.424 2.257) |
| **Age (Ref: 18-25)** | | | |
| 25-29 | -15.079  (-36.561, 6.403) |  | -1.890  (-17.725, 13.946) |
| 30-34 | -40.429***  (-66.016, -14.842) |  | 1.489  (-14.855, 17.833) |
| 35-39 | -21.121  (-50.947, 8.705) |  | 3.001  (-14.353, 20.354) |
| 40-44 | -10.663  (-44.318, 22.992) |  | 7.890  (-8.727, 24.506) |
| 45-49 | -10.989  (-42.484, 20.505) |  | 7.669  (-8.683, 24.020) |
| 50-54 | -27.743*  (-58.831, 3.346) |  | 8.244  (-7.906, 24.395) |
| 55-59 | -12.641  (-42.909, 17.628) |  | 6.710  (-9.600 23.020) |
| **Marital Status (Ref: Never Married)** | | | |
| Currently Married or living together | -12.102  (-35.551, 11.348) |  | -17.688**  (-31.273, -4.103) |
| Divorced/separated/ widowed | 11.699  (-32.693, 56.091) |  | -19.546**  (-37.471 -1.620) |
| **Occupation Status (Ref: Unemployed)** | | | |
| Professional/technical/managerial | 88.994***  (59.863, 118.124) |  | 37.613***  (24.390, 50.836) |
| Clerical | 114.672***  (76.479, 152.865) |  | 1.003  (-23.627, 25.635) |
| Sales and Services | 72.261***  (48.867, 95.655) |  | 30.947***  (19.367, 42.527) |
| Skilled Manual | 67.254***  (45.275, 89.233) |  | 27.548***  (15.926, 39.171) |
| Unskilled Manual | 71.113***  (48.430, 93.797) |  | 32.928***  (21.396, 44.460) |
| Agriculture | 116.525***  (94.795, 138.255) |  | 79.568***  (69.171, 89.964) |
| **Lifestyle Variables** | | | |
| Smoking | -6.559  (-19.163, 6.044) |  | 14.805*  (-1.921, 31.531) |
| Moderate Drinking | -5.389  (-18.545, 7.766) |  | 8.805**  (0.659 16.951) |
| Healthy eating habits | 29.159*  (-2.827, 61.145) |  | 13.591*  (-1.153 28.334) |
| **Health Status compared to a year ago (Ref: Better)** | | | |
| Same | -9.957**  (-18.566, -1.347) |  | -20.852***  (-26.061, -15.644) |
| Poor or worse | -10.646  (-28.306, 7.015) |  | -3.552  (-10.576, 3.472) |
| **Region of residence (Ref: Tiranë)** | | | |
| Berat | -34.061**  (-58.236, -9.885) |  | -39.980***  (-52.425, -27.535) |
| Dibër | -43.702***  (-68.419, -18.984) |  | 13.499**  (1.341, 25.656) |
| Durrës. | -54.594***  (-77.495, -31.693) |  | -88.071***  (-105.887, -70.255) |
| Elbasan | -51.222***  (-82.391, -20.053) |  | -22.917***  (-35.308, -10.526) |
| Fier | -63.228***  (-85.316, -41.140) |  | -26.273***  (-38.236, -14.310) |
| Gjirokastër. | -100.440***  (-134.893, -65.986) |  | -14.245**  (-26.677, -1.812) |
| Korçë | -137.290***  (-166.696, -107.884) |  | -35.497***  (-47.927, -23.068) |
| Kukës | 11.971  (-10.305, 34.247) |  | -54.361***  (-70.045, -38.675) |
| Lezhë | -55.057***  (-80.193, -29.921) |  | -49.199***  (-66.373, -32.024) |
| Shkodër. | -48.092***  (-71.745, -24.437) |  | -66.884***  (-81.668, -52.100) |
| Vlorë | -10.015  (-33.470, 13.439) |  | -9.296  (-21.975, 3.382) |
| **Household Economic Status (Ref: Very Poor and Poor)** | | | |
| Middle | -19.637**  (-36.453, -2.822) |  | -3.543  (-13.363, 6.276) |
| Rich and Very Rich | -3.132  (-21.187, 14.923) |  | -6.667  (-16.939, 3.604) |
| **Household access to Motor vehicle** | -10.381**  (-17.839, -2.924) |  | -1.647  (-6.214, 2.921) |
| **Number of young children in the household (0-4 yrs)** | -22.681**  (-36.980, -8.383) |  | -8.873**  (-16.332, -1.415) |
| **Number of school going children in the household (5-14 yrs)** | -0.134  (-9.857, 9.589) |  | 2.162  (-3.120, 7.444) |
| **Place of residence (Ref: Rural)** | -5.408  (--20.356, 9.539) |  | 10.816**  (1.968, 19.665) |
| **Wald Chi 2 (P > Chi 2)** | 378.20(0.00) |  | 493.12 (0.00) |
| **Log pseudolikelihood** | -14921.809 |  | -30949.493 |
| **Number of observations** | 5,400 |  | 13,652 |
| *** p-value < 0.01, **p-value < 0.05, *p-value <0.10 | | | |
| AME=Average Marginal effects; CI= Confidence interval | | | |
